# Editorial: Ultraviolet Radiation: Friend or Foe for Plants?

**DOI:** 10.3389/fpls.2020.00541

**Published:** 2020-04-29

**Authors:** Laura Llorens, Susanne Neugart, Filip Vandenbussche, Antonella Castagna

**Affiliations:** ^1^Department of Environmental Sciences, Faculty of Sciences, University of Girona, Girona, Spain; ^2^Division of Quality and Sensory of Plant Products, University of Göttingen, Göttingen, Germany; ^3^Laboratory of Functional Plant Biology, Faculty of Sciences, Ghent University, Ghent, Belgium; ^4^Department of Agriculture, Food and Environment, University of Pisa, Pisa, Italy

**Keywords:** UV radiation, plant development, secondary metabolism, signaling, photoprotection, phenols, flavonoids

Ultraviolet (UV) radiation (UV-B, 280–315 nm, and UV-A, 315–400 nm) has important effects on plant growth and development. Despite most of the UV-B radiation is absorbed by the ozone layer, UV, and especially UV-B, has the potential to affect proteins, lipids, and nucleic acids, thereby, representing a potential stressor for plants. However, plants have developed a plethora of acclimatization strategies to avoid excessive UV absorption and to minimize the negative consequences of UV exposure. Therefore, ambient UV levels should be regarded as an environmental and regulatory stimulus, rather than a stressor, having the ability to modulate plant morphology and physiology. This Research Topic gathered six research articles and one review, which contribute to improve our understanding of the mechanisms underlying plant responses to UV radiation, as well as to highlight the potential of this type of radiation for increasing the amount of healthy compounds in vegetables.

The study by Llabata et al. provide evidence, using *Arabidopsis thaliana* mutants, that a serine/threonine-protein kinase called GCN2 acts as a negative regulator of UV-B responses. GCN2 plays a key role in the modulation of protein biosynthesis in response to different environmental stresses. Llabata et al. found that *gcn2* mutants showed increased tolerance toward chronic exposure to UV-B which correlated with a reduced formation of cyclobutane pyrimidine dimers. They hypothesized that GCN2 might be involved in the transcriptional repression of the energy demanding biosynthesis of stress protective compounds.

*Arabidopsis thaliana* has been until now a useful model species to investigate UV-B responses mediated by the UV-B specific receptor UVR8. However, the review by Tossi et al. shows that many mechanisms described in this species differ from other species. While in some species, such as *Arabidopsis*, UVR8 expression is constitutive under UV-B exposure, in others it varies in a tissue- and development-dependent way. The authors provide evidence of UVR8 being involved in other developmental and stress processes unrelated to UV-B, suggesting that ABA could play a key role in the induction of UVR8 expression.

The study of protective strategies against excessive photosynthetically active (PAR) and UV radiation is especially interesting in bryophytes, since they are considered the first true plants that colonized land. Soriano et al. tried to elucidate to what extent the response strategies to sun and shade conditions of the liverworts *Marchantia polymorpha* and *Jungermannia exsertifolia* and the moss *Fontinalis antipyretica* were influenced by PAR and UV wavelengths. *M. polymorpha* was the most responsive species, with sun plants showing responses directed to reduce light absorption, alleviate overexcitation and increase photoprotection. Both PAR and UV were responsible for the increase in the xanthophyll index (related to non-photochemical dissipation of excess energy), while increased contents of apigenin and luteolin derivatives (which can act as antioxidants and/or UV screens) were only determined by UV radiation. In contrast, increases in the sclerophylly index, as well as reductions in the chlorophyll *a*+*b* content, in sun samples seemed to be mediated only by PAR.

Another photoprotective mechanism observed in multiple plant species is the movement of chloroplasts, which is induced by blue light through phototropins (blue/UV-A light photoreceptors). In weak light, chloroplasts accumulate perpendicular to the direction of incident light in order to maximize light absorption. In contrast, under strong blue light, chloroplasts position at cell walls parallel to the direction of incident light to avoid photodamage. Studying *Arabidopsis thaliana* leaves, Hermanowicz et al. found that UV-B induces chloroplast movements in a phototropin-dependent manner, with UVR8 not being involved in this response. They suggest that perception of UV-B by phototropins may be important under tree canopies, where UV-B to UV-A ratios are higher than under sunlight, since it may contribute to the induction of chloroplast accumulation and thus to the optimization of photosynthesis.

In the last decades, there has been an increasing interest in the practical applications of UV to produce sustainable and high-quality food, due to its capacity to stimulate the accumulation of healthy compounds, such as certain flavonoids. In this line, Blancquaert et al. followed the evolution of flavonoids during the ripening of Cabernet Sauvignon (*Vitis vinifera*) grapes over two seasons under altered temperature and light conditions. Flavonoids contribute to the potential beneficial effects of wine on human health, with flavonols being one of the main flavonoid groups identified. Blancquaert et al. showed that reduced UV-B light in the bunch zone decreased skin flavonol biosynthesis, highlighting the importance of this type of radiation for flavonol production in grapes.

UV-A radiation can also promote the accumulation of health-promoting compounds, such as flavonoids. However, commonly used UV-A lamps encompassed a wide range of UV-A wavelengths and did not allow to identify those wavelengths responsible for the observed effects. Because of this, Lee et al. grew plants of kale (*Brassica oleracea* var. *acephala*) under two types of UV-A LEDs (370 and 385 nm) to evaluate their effects on the growth and production of antioxidant phenolic compounds. They found that UV-A irradiation, especially at 385 nm, increased plant growth, as well as leaf total phenolic content and antioxidant capacity. Brazaityté et al. came to a similar conclusion working with mustard microgreens (*Brassica juncea* cv. “Red Lion”) irradiated with UV-A LEDs at different wavelengths (366, 390, and 402 nm) and durations (10 and 16 h). They found that the concentration of total phenols and α-tocopherol mostly increased at 402 nm, although increases were also detected at 390 and/or 366 nm. Positive effects of specific UV-A wavelengths on lutein/zeaxanthin and β-carotene contents, as well as on the accumulation of mineral elements (except Fe), were also reported. These results support the idea that UV-A LEDs can be a useful tool to improve the nutritional quality of vegetables.

In conclusion, evidence highlights the important role of UV radiation in the modulation of plant physiology and metabolism. However, there are still basic open questions, as well as many applications and methodological gaps to fill. Since most of the responses to UV radiation seem to be species-specific and dose-dependent, a wider range of species and UV doses need to be investigated in order to successfully answer these questions.

## Author Contributions

LL wrote the first draft of the article. All the other authors made a substantial, intellectual contribution to the work, and approved it for publication.

## Conflict of Interest

The authors declare that the research was conducted in the absence of any commercial or financial relationships that could be construed as a potential conflict of interest.

